# Mental health screening in women with polycystic ovary syndrome: protocol for a systematic review and meta-analysis

**DOI:** 10.1186/s13643-026-03269-9

**Published:** 2026-07-16

**Authors:** Rajalakshmi Rajendran, Marva Abdul Khader, Priya Shivanna, Prathiksha M. Shet, Niyam Hegde, Deeya Karkera, Girish Thunga

**Affiliations:** Department of Pharmacy Practice, Manipal College of Pharmaceutical Sciences, Manipal Academy of Higher Education, Manipal, Karnataka 576104 India

**Keywords:** Mental health, Polycystic ovary syndrome, Psychological well-being, Quality of life, Women’s health

## Abstract

**Background:**

Polycystic ovary syndrome (PCOS) is a common endocrine disorder affecting women of reproductive age and is reported frequently with mental health disorders. This systematic review aims to synthesize the available evidence on mental health screening and its potential role in identifying and addressing mental health conditions among women with PCOS.

**Methods:**

This systematic review will be conducted according to JBI methodology for systematic reviews of effectiveness. The search strategy will follow the Peer Review of Electronic Search Strategies (PRESS) checklist. Literature searches will be conducted in the Cochrane Library, PubMed/MEDLINE, Scopus, Embase, CINAHL, Web of Science, and PsycINFO databases, as well as relevant grey literature sources. Studies involving women with PCOS that report the use of mental health screening tools or assessment of mental health outcomes will be eligible for inclusion. Experimental, quasi-experimental, and observational studies will be considered. Two independent reviewers will perform study selection and data extraction using predefined eligibility criteria and standardized forms, with disagreements resolved through discussion or consultation with a third reviewer. Methodological quality will be assessed using the JBI critical appraisal tools appropriate to the study design. Where sufficient data are available, a meta-analysis will be conducted; otherwise, findings will be synthesized narratively. The certainty of evidence will be evaluated using the Grading of Recommendations Assessment, Development and Evaluation (GRADE) approach.

**Discussion:**

This systematic review will provide a comprehensive synthesis of the available evidence on mental health screening among women with PCOS. The findings may inform clinical practice, support evidence-based guideline development, and identify priorities for future research in the holistic management of PCOS.

**Systematic review registration:**

PROSPERO CRD420251240567

**Supplementary Information:**

The online version contains supplementary material available at https://doi.org/10.1186/s13643-026-03269-9.

## Background

Polycystic Ovary Syndrome (PCOS) is a complex endocrine disorder characterized by hyperandrogenism, irregular ovulation, and morphological changes in the ovaries, affecting approximately 8–13% of women of reproductive age worldwide, with a recent 2025 meta-analysis estimating a global prevalence of 9.2% [1]. Genetic predisposition represents a major risk factor for PCOS, while environmental and lifestyle factors also contribute significantly to its development [2]. Diagnostic criteria for PCOS are primarily based on the Rotterdam, Androgen Excess Society (AES), and National Institutes of Health (NIH) guidelines [1, 2]. According to the Rotterdam criteria, PCOS is diagnosed when at least two of the following features are present after excluding other aetiologies: clinical or biochemical hyperandrogenism, oligo-anovulation, and polycystic ovarian morphology. Four distinct phenotypes of PCOS (A–D) have been identified, with phenotype A representing the most severe clinical form and phenotype D the mildest [3]. Beyond its reproductive manifestations, PCOS is associated with substantial impairment in quality of life, driven by physical changes such as acne, hirsutism, alopecia, and weight gain, as well as metabolic and reproductive complications including insulin resistance, type 2 diabetes, dyslipidaemia, obesity, infertility, and emotional vulnerability [4].

Mental health disorders constitute a significant global public health burden and arise from complex interactions among biological, psychological, and social determinants. Mental and physical health are closely interrelated and exert reciprocal effects on one another. In individuals with PCOS, this interaction is particularly pronounced, with numerous studies demonstrating a strong association between PCOS and psychological morbidity, particularly affecting the psychological and social domains of quality of life [5, 6]. Clinical differentiation between PCOS-related symptoms and primary psychiatric disorders is often challenging due to considerable symptom overlap, and this complexity is further heightened by the fact that certain psychotropic medications, particularly antipsychotics, may induce PCOS-like features such as menstrual irregularities, amenorrhea, and weight gain. Among women with PCOS, depression and anxiety are the most frequently reported psychological conditions, with prevalence estimates of approximately 47.7% and 88%, respectively [7, 8]. Additional psychosocial concerns commonly reported in this population include diminished libido, increased stress, loneliness, body image dissatisfaction, and reduced self-esteem [9, 10]. PCOS presents a unique clinical context in which the psychological burden is closely linked to its reproductive, endocrine, metabolic, and cosmetic manifestations including infertility and body image concerns, leading to a distinct pattern of psychological morbidity. The growing recognition of these psychological comorbidities has shifted the clinical management of PCOS from focusing solely on reproductive and metabolic features to adopting a more holistic, patient-centered approach. Reflecting this shift, the 2023 International Evidence-Based Guideline for the Assessment and Management of PCOS recommends that healthcare professionals incorporate routine mental health screening into standard PCOS care, including assessment of symptom severity, associated risk factors, and the presence of self-harm or suicidal ideation, with referral for specialist mental health care when moderate to severe symptoms are identified [11].

However, existing systematic reviews provide limited guidance regarding the screening tools used, their implementation in clinical practice, and the integration of mental health screening into routine PCOS care. Consequently, clinicians have limited synthesized evidence to inform the selection and implementation of appropriate mental health screening approaches in women with PCOS. This gap in the evidence synthesis highlights the need for a comprehensive systematic review focused specifically on mental health screening in individuals with PCOS to support early identification, guide clinical decision-making, and improve long-term outcomes.

Accordingly, this review is intended to inform healthcare professionals involved in the management of PCOS, including primary care physicians, gynecologists, endocrinologists, reproductive medicine specialists, and other clinicians responsible for ongoing care. By synthesizing the available evidence on mental health screening practices, this review aims to identify feasible screening approaches, inform clinical decision-making, and highlight areas requiring further research to improve the integration of mental health care into routine PCOS management.

## Objectives

### Primary objective

To assess the effect of routine mental health screening, compared with no screening or usual care, on mental health outcomes among women diagnosed with PCOS.

### Secondary objectives

- To examine the effect of routine mental health screening on the detection of mental health conditions among women with PCOS.
- To identify the types of mental health outcomes (e.g., depression, anxiety, and quality of life) assessed in studies evaluating routine mental health screening among women with PCOS.
- To identify gaps in the existing evidence on mental health screening among women with polycystic ovary syndrome (PCOS) and highlight priorities for future research.

### Inclusion and exclusion criteria

The study eligibility criteria follow the Population, Intervention/Exposure, Comparator, and Outcome (PICO) framework as summarized in Table 1 [12]. Additional inclusion and exclusion criteria were established to ensure that the selected studies are directly relevant to the review question and meet the predefined eligibility requirements.

**Table 1:** Inclusion and exclusion criteria for study selection based on the PICOS framework

Inclusion criteria	Exclusion criteria
Population (P): women diagnosed with PCOS within the reproductive age group, including adolescents and adults, based on recognized diagnostic criteria (e.g., Rotterdam criteria) who do not have a previously established diagnosis of a mental health disorder at the time of screening	
Women of reproductive age with PCOS, defined as individuals clinically diagnosed using recognized diagnostic criteria (e.g., Rotterdam, NIH, or AE-PCOS Society criteria) or with a confirmed diagnosis documented in scientific literature	Women without a clinical or research diagnosis of PCOS (e.g., ovarian cysts from other causes) unless a subgroup with confirmed PCOS is separately reported
Adolescents and adults: typically 11–19 (for adolescent as per WHO years (or puberty onset) and reproductive age group for adults	Pregnant women at the time of screening, to avoid confounding effects of pregnancy-related hormonal and psychological changes
	Women with other untreated endocrine conditions that can mimic PCOS or independently cause psychiatric symptoms (e.g., untreated Cushing’s syndrome, hyperprolactinaemia, thyroid storm), unless PCOS is a co diagnosis and data are separable
Intervention(s) or exposure(s): routine mental health screening using validated screening tools administered in clinical or community settings	
Studies in which mental disorders as described by the WHO International Classification of Diseases 11th Revision (ICD-11) [13] screening implemented for women with PCOS, including but not limited to assessment for depression, Anxiety, bipolar disorder, eating disorders, post-traumatic stress disorder, schizophrenia, disruptive behavior and dissocial disorders, neurodevelopmental disorders and other psychiatric conditions relevant to PCOS populations	Studies that do not include any form of mental health screening or psychological assessment
Use of a recognised or validated mental-health screening tool, such as PHQ-9, GAD-7, HADS, BDI, DASS-21, or similar structured tools (non-validated tools may be included only if clearly described and comparable.)	Studies focusing solely on treatment or management of mental health conditions in women with PCOS, without screening as a component (e.g., therapy, medication, lifestyle intervention alone)
Exposures comparing screened vs. non-screened groups, or evaluating mental-health outcomes following introduction of screening practices	Screening or assessment tools that are not defined, recognized, or validated, and where methodology cannot be reliably interpreted
	Studies evaluating general well-being or quality-of-life only, without screening for specific mental health conditions
	Interventions where outcomes are unrelated to mental-health identification, such as metabolic, reproductive, or dermatological outcomes only
Comparator(s) or control(s): no screening or usual care (i.e., standard clinical practice without structured mental health screening)	
The review may compare routine mental health screening with standard care/no screening where evidence exists. If direct comparative studies (routine screening versus no screening or usual care) are unavailable or limited, findings from non-comparative studies will be synthesized descriptively to characterize mental health screening practices, screening tools, detection rates, and reported outcomes. Such evidence will not be used to infer the comparative effectiveness of routine mental health screening but will be considered to identify current practices, implementation strategies, and evidence gaps	
Outcome(s)- Detection/identification of mental health conditions; changes in mental health outcomes (e.g., depression, anxiety, quality of life); and any identified evidence gaps for future research	
Studies will be included if they report at least one of the following outcomes related to the effectiveness of routine mental health screening: • Changes in mental health status, including depression, anxiety, or quality of life, measured using validated instruments • Rate of detection of clinically significant mental health conditions • Initiation of appropriate mental health care • Adverse effects associated with screening (if any)	Studies reporting only the prevalence of mental health conditions in women with PCOS without assessing the impact of screening
	Studies focused solely on the validation or psychometric properties of screening tools without evaluating their impact on clinical outcomes
	Studies that do not report outcomes related to the effectiveness of screening (e.g., mental health outcomes, detection rates, treatment initiation)
Study design	
This review will include experimental and quasi-experimental study designs, including randomized controlled trials, cluster randomized trials, and non-randomized controlled trials. In addition, observational analytical studies with a comparator group, including prospective and retrospective cohort studies, will be considered	Studies without a comparison group, including cross-sectional studies, case-control studies, case series, and case reports, will be excluded

### Context

This review will include studies conducted in clinical, community, or research settings where women with polycystic ovary syndrome (PCOS) are assessed for mental health conditions. There are no restrictions based on country or healthcare system, provided the mental health screening method is described and applicable to women with polycystic ovary syndrome (PCOS). 

## Methods

The proposed systematic review will be conducted in accordance with JBI methodology for systematic reviews of effectiveness [14] and reported in line with the Preferred Reporting Items for Systematic Reviews and Meta-Analyses (PRISMA 2020) guidelines [15]. The systematic review protocol was developed and reported according to the Preferred Reporting Items for Systematic Review and Meta-Analysis Protocols (PRISMA-P) 2015 statement [16]. Additionally, the study is registered with International Prospective Register of Systematic Reviews (PROSPERO) with number CRD420251240567. The methodology is described as follows:

### Step 1: Identifying the research question

The main research question is “Does routine mental health screening, compared to no screening or usual care, improve mental health outcomes among women diagnosed with PCOS?”.

The secondary research questions include the following.

- What is the effect of routine mental health screening on the detection of mental health conditions in women with polycystic ovary syndrome (PCOS)?
- What types of mental health outcomes (e.g., depression, anxiety, and quality of life) are assessed following routine mental health screening in women with polycystic ovary syndrome (PCOS)?
- What gaps exist in the current evidence on mental health screening among women with polycystic ovary syndrome (PCOS), and what are the priorities for future research?

### Step 2: Conducting a relevant literature search

A preliminary search of PROSPERO, the Cochrane Database of Systematic Reviews, JBI Evidence Synthesis, and PubMed was conducted in January 2026 to identify existing systematic reviews and review protocols on the topic. No published or ongoing reviews addressing the same research question were identified.

The search strategy has been developed using the PICO framework and combines controlled vocabulary terms (e.g., MeSH and Emtree terms) with free-text keywords related to PCOS, mental health conditions, and mental health screening. The search strategy will be adapted for each database using appropriate field tags, database-specific indexing, and Boolean operators to maximize the retrieval of relevant studies while minimizing irrelevant results. To validate the search strategy, a benchmark set of key studies known to be relevant to the review question will be identified. Pilot searches will be conducted to determine whether these studies are retrieved by the search strategy. If relevant studies are not identified, the search strategy will be refined by modifying search terms, synonyms, Boolean operators, or field codes as appropriate. All modifications and their rationale will be documented to enhance transparency and reproducibility.

The search strategy has been developed by two authors (GT and RR) in consultation with an experienced systematic review librarian. Before implementation, the strategy will be peer-reviewed in accordance with the Peer Review of Electronic Search Strategies (PRESS) 2015 Guideline Statement [17]. The final comprehensive literature search will be conducted by GT and RR and will cover each database from its inception to the date of the final search. The exact dates on which the searches are conducted will be reported in the completed systematic review. The comprehensive literature search will be conducted across multiple electronic databases, including PubMed, Embase, Scopus, CINAHL, APA PsycINFO, Web of Science Core Collection, and the Cochrane Library. These databases were selected to ensure broad coverage of biomedical, reproductive health, nursing, psychological, and mental health literature relevant to women with PCOS. The search process and results will be reported in accordance with the Preferred Reporting Items for Systematic Reviews and Meta-Analyses Search Extension (PRISMA-S) to ensure transparent and reproducible reporting [18]. The complete search strategies for all databases, trial registries, and other information sources are provided in Supplementary file 1.

To identify ongoing and unpublished studies, trial registries including the International Clinical Trials Registry Platform (ICTRP) and the Clinical Trials Registry–India (CTRI) will be searched. Grey literature searches will include government reports, conference proceedings, institutional repositories, university theses, digital repositories such as Shodhganga, and policy briefs from major health agencies, including the World Health Organization (WHO) and the National Centre for Disease Control (NCDC), to capture evidence that may not be published in traditional journals. Additional studies will be identified through backward and forward citation tracking of included studies and by searching supplementary sources such as Google Scholar to identify potentially relevant studies that may not be indexed in the selected databases.

No language restrictions will be applied during the search process. Studies published in languages other than English will be translated using Microsoft Translator. Titles and abstracts will be screened using translated text, and full-text articles meeting the eligibility criteria will be translated in full. Where feasible, bilingual reviewers will verify translated content to ensure accuracy and consistency.

### Step 3: Study selection and screening

All records retrieved from the electronic database searches will be exported to Zotero 7.0.30 for reference management and duplicate removal. Following deduplication, the unique records will be imported into Rayyan for study screening. Before initiating the study selection process, all reviewers will undergo a structured training protocol to ensure consistency in applying the inclusion and exclusion criteria (Table 1). This training will include familiarization with the eligibility criteria and a pilot screening exercise on a subset of studies for blinded and independent selection. Two reviewers (RR and MAK) will independently and blindly screen a randomly selected sample of 50 titles and abstracts to assess the clarity and consistency of the eligibility criteria. Discrepancies will be discussed and resolved through consensus, with modifications to the screening criteria made where necessary before commencing the full screening.

Two reviewers (NH and DK) will independently conduct both the title and abstract screening, as well as the full-text screening, ensuring their assessments are made without prior discussion.

The screening process will follow a two-stage approach to efficiently manage and evaluate the search results:

(i) Title and abstract screening: in the first stage, we will screen the titles and abstracts of the retrieved articles. Human reviewers will assess each entry for relevance to our review question based on our inclusion criteria. Articles that do not meet the criteria will be excluded at this stage.
(ii) Full-text screening: in the second stage, we will perform a full-text review of the articles that passed the title and abstract screening. Human reviewers will comprehensively evaluate the content of each study to confirm its eligibility for inclusion in the review. Any articles that do not meet the established criteria will be excluded.

Any disagreements during either stage will be resolved through discussion and consensus. If consensus cannot be reached, a third reviewer (GT) will adjudicate.

Where required information is unavailable, the corresponding author will be contacted by email on up to two occasions, approximately two weeks apart. If no response is received within four weeks after the initial contact, the study will be assessed using the available information, and, where critical information remains unavailable, it may be excluded with the reason documented.

The study selection process will be reported using a PRISMA 2020 flow diagram detailing the number of records identified, screened, excluded, and included at each stage.

### Step 4: Data extraction

The data from the primary research studies will be extracted using a standardized data extraction form developed specifically for this review based on the review objectives and relevant literature. The data extraction form will be piloted on a sample of included studies and refined, where necessary, before full data extraction. Key elements for extraction, such as demographic details, outcome measures, and risk of bias indicators, will be carefully selected to address the research questions and support a comprehensive analysis. The extraction process will follow a multi-stage approach, starting with independent data extraction by two extractors, followed by a reconciliation phase to resolve any discrepancies. This two-step process enhances reliability by fostering discussion and consensus among extractors.

During the data extraction phase, two extractors (PS and PMS) will independently work on a predetermined set of studies to ensure unbiased extraction. Their outputs will be compared to identify discrepancies, which will then be resolved through discussion. In cases of significant differences, a senior researcher (GT) may be consulted to reach a consensus.

### Step 5: Methodological quality assessment

The methodological quality of the included studies will be assessed using JBI critical appraisal tools according to the study design of the included studies [19]. Specifically, JBI critical appraisal checklist for randomized controlled trials will be used for RCTs, while other study designs will be using corresponding JBI checklists. Each study will be assessed across the methodological domains such as selection bias, measurement of outcomes, statistical analysis, and confounding factors.

Two assessors (PS and PMS) independently assess the methodological quality of the included studies, and any disagreement will be resolved by mutual consensus or in consultation with a third assessor (GT). Each item in the checklist will be rated as “yes,” “no,” “unclear,” or “not applicable” and the assessment results will be visually presented using Microsoft Excel 2016. Studies will not be excluded based solely on methodological quality; however, the appraisal results will be considered when interpreting the findings of the review.

### Data synthesis and reporting

The analysis procedure to address the research questions involves systematic steps. A narrative synthesis will be applied for all included studies. A meta-analysis will be performed based on the availability of sufficient homogenous data in terms of population, intervention, comparator, and outcomes. For dichotomous outcomes (e.g., detection of mental health conditions, initiation of treatment), effect sizes will be expressed as risk ratios (RR) or odds ratios (OR) with 95% confidence intervals. For continuous outcomes (e.g., depression or anxiety scores, quality of life), mean differences (MD) or standardized mean differences (SMD) will be used. The primary analysis will aggregate extracted data, and a suitable effect model (fixed or random effect model) to address heterogeneity and to calculate overall effect sizes will be employed. In the review, conclusions will be drawn based on the statistical significance of effect measures rather than a predetermined minimum effect size (*p* < 0.05). Heterogeneity will be assessed using the I^2^ statistic, where thresholds of 0–25%, 25–50%, and 50–75% indicate low, moderate, and high heterogeneity, respectively.

In the synthesis process, there will be one primary researcher (RR) who will be responsible for synthesizing the extracted data. Additionally, a subject expert, methodologist, and statistician will be included to provide input and ensure the synthesis is comprehensive and robust. The synthesist will first conduct the analyses independently, applying their own interpretations and conclusions to the data. After completing the individual synthesis, the findings will be discussed among the research team and any differences in the interpretations reconciled. If significant divergences arise during the reconciliation, the team may consult with the third/subject expert to provide additional insight and guidance. If meta-analysis is not appropriate due to heterogeneity or insufficient data, findings will be presented using a structured narrative synthesis.

Where sufficient data are available, subgroup analyses will be conducted based on type of mental health condition, type of screening tool used, healthcare setting, age group, and geographical context. These analyses aim to explore potential sources of heterogeneity and identify factors that may influence the effectiveness of routine mental health screening in women with PCOS. Similarly, when sufficient data are available, sensitivity analysis will be conducted to assess the robustness of the findings either by excluding studies having high risk of bias or by leave-one-out analysis, where applicable. The results of sensitivity analyses will be compared with the primary analysis to determine the stability and reliability of the findings.

To assess the publication bias, visual inspection of funnel plots will be performed and if asymmetry is detected, potential causes such as heterogeneity, methodological quality, or selective reporting will be explored. Additionally, Egger’s and Begg’s test will be performed to statistically evaluate funnel plot asymmetry and assess the likelihood of publication bias in the literature [20]. If significant publication bias is detected, methods such as trim-and-fill analysis will be applied to estimate the number of missing studies and adjust the overall effect size accordingly.

The methodological limitations of the included studies will be reported systematically to ensure transparency and critical appraisal of the findings. These limitations will be summarized in a narrative format, highlighting their potential impact on the interpretation of results.

This gap identification will provide insights into areas that require further research to support evidence-based management of PCOS focusing on mental health.

### Strength of evidence assessment

The overall strength (certainty) of the evidence for each outcome will be assessed using the Grading of Recommendations Assessment, Development and Evaluation (GRADE) approach [21]. The certainty of the evidence will be evaluated across five domains: risk of bias, inconsistency, indirectness, imprecision, and publication bias. The quality of evidence will be rated as high, moderate, low, or very low. Two assessors (RR and MAK) will independently perform the GRADE assessment, with any disagreements resolved through discussion or consultation with a third assessor (GT). The results of the GRADE assessment will be presented in a Summary of Findings (SoF) table to facilitate interpretation of the review findings.

## Discussion

The proposed systematic review aims to underscore the need for routine mental health screening in women diagnosed with PCOS. It will also highlight gaps in the existing knowledge regarding the prevalence and management patterns of women with PCOS. This review represents the first step toward the invisible struggle of mental health issues faced by women with PCOS that needs to be recognized and resolved. Furthermore, the findings may create awareness about the burden of psychiatric disorders and their associated challenges, guiding healthcare policymakers and stakeholders to address these issues effectively in a cohort of women of reproductive age.

The review is likely to be limited by heterogeneity in polycystic ovary syndrome (PCOS) diagnostic criteria and mental health screening tools across studies, which may restrict comparability. Most evidence relies on self-reported screening instruments rather than diagnostic interviews, potentially overestimating prevalence.

### Ethics and dissemination

This systematic review will synthesize data from previously published studies and will not involve the collection of primary data from human participants. Therefore, ethical approval and informed consent are not required.

The findings of this review will be disseminated through publication in a peer-reviewed journal and presentation at national and international scientific conferences. The review is expected to provide evidence to inform clinical practice, future research, and healthcare policy regarding the study topic.

## Supplementary Information

Supplementary Material 1. Supplementary file S1: Detailed search strategy for all electronic databases.

## Data Availability

No datasets were generated or analysed during the current study.

## Abbreviations

PCOS: Polycystic ovary syndrome
AES: Androgen Excess Society
NIH: National Institute of Health
SCL-90: Symptom checklist 90 scoring
PHQ-9: Patient health questionnaire
GAD: Generalized anxiety disorder scale
HAD: Hospital anxiety and depression scale
PCOSQ: Polycystic Ovarian Syndrome Health-Related Quality of Life Questionnaire
SF-36: Short form health survey-36
JBI: Joanna Briggs Institute
CINAHL: Cumulative Index to Nursing and Allied Health Literature
WOS: Web of Science
PRISMA-P 2015: Preferred Reporting Items for Systematic Reviews and Meta-Analyses
PRISMA-S: Preferred Reporting Items for Systematic Reviews and Meta-Analyses literature Search extension
PROSPERO: International Prospective Register of Systematic Reviews
ICTRP: International Clinical Trials Research Platform
CTRI: Clinical Trials Registry-India
PRESS: Peer Review of Electronic Search Strategies
WHO: World Health Organization
NCDC: National Centre for Disease Control
PRISMA-P: Preferred Reporting Items for Systematic Reviews and Meta-Analyses
