# Supplementary material for: Mental health screening in women with polycystic ovary syndrome: protocol for a systematic review and meta-analysis
Source: Syst Rev. 2026 Jul 16;15:266. doi: 10.1186/s13643-026-03269-9 (PMC13656675; doi:10.1186/s13643-026-03269-9)
Supplement: Supplementary file 1 [file 13643_2026_3269_MOESM1_ESM.docx]

The detailed search strategy in different databases

Date searched: 12-02-2026

A. PubMed Search strategy

| Search | Query | Items found |
| --- | --- | --- |
| #1 | ((((((((((((((((((Ovary Syndrome, Polycystic) OR (Syndrome, Polycystic Ovary)) OR (Polycystic Ovarian Syndrome)) OR (Ovarian Syndrome, Polycystic)) OR (Polycystic Ovary Syndrome 1)) OR (Sclerocystic Ovarian Degeneration)) OR (Ovarian Degeneration, Sclerocystic)) OR (Sclerocystic Ovary Syndrome)) OR (Stein-Leventhal Syndrome)) OR (Stein Leventhal Syndrome)) OR (Syndrome, Stein-Leventhal)) OR (Sclerocystic Ovaries)) OR (Ovary, Sclerocystic)) OR (Sclerocystic Ovary)) OR (polycystic ovary syndrome[MeSH Terms])) OR (polycystic ovary*[Title/Abstract])) OR (PCOD[Text Word])) OR (PCOS[Text Word])) OR (polycystic ovar*) | 30,110 |
| #2 | (((((((((((mental disorders[MeSH Terms]) OR (association, mental health[MeSH Terms])) OR (Anxiety[MeSH Terms])) OR (clinical depression[MeSH Terms])) OR (bipolar disorder[MeSH Terms])) OR (eating disorders[MeSH Terms])) OR (acute post traumatic stress disorder[MeSH Terms])) OR (Schizophrenia[MeSH Terms])) OR (Neurodevelopmental disorders[MeSH Terms])) OR (Mental*[Text Word])) OR (Psychia*[Title/Abstract])) | 21,92,302 |
| #3 | (((mass screening[MeSH Terms]) OR (screening[Title/Abstract])) OR (screen*[Title/Abstract])) OR (detect*[Title/Abstract]) | 41,29,480 |
| #4 | ((#1) AND (#2)) AND (#3) | 174 |

B. SCOPUS Search Strategy

| Search | Query | Items found |
| --- | --- | --- |
| #1 | ( ALL ( "Ovary Syndrome, Polycystic" ) OR ALL ( "Syndrome, Polycystic Ovary" ) OR ALL ( "Polycystic Ovarian Syndrome" ) OR ALL ( "Ovarian Syndrome, Polycystic" ) OR ALL ( "Polycystic Ovary Syndrome 1" ) OR ALL ( "Sclerocystic Ovarian Degeneration" ) OR ALL ( "Ovarian Degeneration, Sclerocystic" ) OR ALL ( "Sclerocystic Ovary Syndrome" ) OR ALL ( "Stein-Leventhal Syndrome" ) OR ALL ( "Stein Leventhal Syndrome" ) OR ALL ( "Syndrome, Stein-Leventhal" ) OR ALL ( "Sclerocystic Ovaries" ) OR ALL ( "Ovary, Sclerocystic" ) OR ALL ( "Sclerocystic Ovary" ) OR TITLE-ABS-KEY ( "polycystic ovary syndrome" ) OR TITLE-ABS-KEY ( "polycystic ovar*" ) ) | 49,267 |
| #2 | ( ALL ( "mental disorders" ) OR ALL ( "association, mental health" ) OR ALL ( "Anxiety" ) OR ALL ( "clinical depression" ) OR ALL ( "bipolar disorder" ) OR ALL ( "eating disorders" ) OR ALL ( "acute post-traumatic stress disorder" ) OR ALL ( "Schizophrenia" ) OR ALL ( "Neurodevelopmental disorders" ) OR ALL ( "Mental*" ) OR ALL ( "Psychia*" ) ) | 6,976,164 |
| #3 | ( ALL ( "mass screening" ) OR ALL ( "screening" ) OR ALL ( "screen*" ) OR ALL ( "detect*" ) ) | 19,979,055 |
| #4 | ((#1) AND (#2)) AND (#3) | 1780 |

C. Embase Search Strategy

| Search | Query | Items found |
| --- | --- | --- |
| #1 | 'ovary polycystic disease'/exp OR 'ovary polycystic disease' | 46480 |
| #2 | 'mental disease'/exp OR 'mental disease' | 3390848 |
| #3 | 'screening'/exp OR 'screening' | 1653713 |
| #4 | #1 AND #2 AND #3 | 184 |

D. Cochrane Search Strategy

| Search | Query | Items found |
| --- | --- | --- |
| #1 | ("polycystic ovarian disease") OR ("polycystic ovary syndrome") (Word variations have been searched) | 4750 |
| #2 | ("mental health") OR ("mental disease") (Word variations have been searched) | 50798 |
| #3 | (screening) or (assessment) or (test) or (diagnosis) (Word variations have been searched) | 846423 |
| #4 | #1 AND #2 AND #3 | 83 |

E. CINAHL

| Search | Query | Items found |
| --- | --- | --- |
| #1 | (mental health or mental illness or mental disorder or psychiatric illness) | 4750 |
| #2 | (polycystic ovary syndrome or pcos or polycystic ovarian syndrome or polycystic ovaries) | 50798 |
| #3 | (screening or assessment or test or diagnosis) | 102 |
| #4 | #1 AND #2 AND #3 | 549 |

F. Web of Science

| Search | Query | Items found |
| --- | --- | --- |
| #1 | TS=("polycystic ovary syndrome*" OR "polycystic ovarian syndrome*" OR PCOS OR PCOD OR "Stein-Leventhal syndrome*" OR "sclerocystic ovar*") | 35833 |
| #2 | TS=("mental health" OR "mental disorder*" OR psychiatric OR psychiatry OR depression OR depressive OR anxiety OR anxious OR bipolar OR schizophrenia OR psychosis OR "eating disorder*" OR "neurodevelopmental disorder*") | 1968306 |
| #3 | TS=(screening OR screen* OR detect* OR identification OR assessment) | 11204035 |
| #4 | #1 AND #2 AND #3 | 519 |

G. PsycINFO

| Search | Query | Items found |
| --- | --- | --- |
| #1 | ("polycystic ovary syndrome*" OR "polycystic ovarian syndrome*" OR PCOS OR PCOD OR "Stein-Leventhal syndrome*" OR "sclerocystic ovar*") | 756 |
| #2 | (DE "Mental Health" ORDE "Mental Disorders" OR DE "Anxiety" OR DE "Depression" OR DE "Bipolar Disorder" OR DE "Eating Disorders" OR DE "Schizophrenia" OR TX ("mental health" OR psychiatric OR psychiatry OR depression OR depressive OR anxiety OR anxious OR bipolar OR psychosis OR schizophrenia OR "eating disorder*")) | 242515 |
| #3 | (screen* OR screening OR detect* OR identif* OR assess* OR diagnos*) | 46610 |
| #4 | #1 AND #2 AND #3 | 16 |

International Clinical Trials Registry Platform (ICTRP)

Search date: 14-02-2026

Search terms:

- "polycystic ovary syndrome"
- PCOS
- "mental health"
- depression
- anxiety
- screening

Search strategy:

("polycystic ovary syndrome" OR PCOS) AND ("mental health" OR depression OR anxiety) AND screening

Clinical Trials Registry–India (CTRI)

Search date: 14-02-2026

Search terms:

- "polycystic ovary syndrome"
- PCOS
- "mental health"
- depression
- anxiety
- screening

Search strategy:

("polycystic ovary syndrome" OR PCOS) AND ("mental health" OR depression OR anxiety) AND screening

Grey Literature Sources

Google Scholar

Search date: [DD Month YYYY]

Search string:

("polycystic ovary syndrome" OR PCOS) AND ("mental health" OR depression OR anxiety) AND screening

Screening approach:
The first 200 results sorted by relevance were screened.

Shodhganga

Search terms:

- "polycystic ovary syndrome" AND mental health
- PCOS AND depression
- PCOS AND anxiety
- PCOS AND screening

WHO Publications and Reports

Search terms:

- "polycystic ovary syndrome" AND mental health
- PCOS AND screening

ResearchGate

Search terms:

- "polycystic ovary syndrome" AND mental health screening
- PCOS AND depression screening
